# Metformin: An Old Drug with New Applications

**DOI:** 10.3390/ijms19102863

**Published:** 2018-09-21

**Authors:** Joseph Zhou, Scott Massey, Darren Story, Lixin Li

**Affiliations:** 1 College of Medicine, Central Michigan University, Mount Pleasant, MI 48859, USA; zhou1jy@cmich.edu; 2 Physician Assistant Program, College of Health Professions, Central Michigan University, Mount Pleasant, MI 48859, USA; masse2sl@cmich.edu; 3 Program in Neuroscience, Central Michigan University, Mount Pleasant, MI 48859, USA; story1dt@gmail.com

**Keywords:** metformin, NAFLD, inflammation, metabolic syndrome, ER stress

## Abstract

Metformin is a biguanide drug that has been used to treat type 2 diabetes mellitus for more than 60 years. The United Kingdom Prospective Diabetic Study (UKPDS) has shown metformin to improve mortality rates in diabetes patients, and recent studies suggest metformin has additional effects in treating cancer, obesity, nonalcoholic fatty liver disease (NAFLD), polycystic ovary syndrome (PCOS), and metabolic syndrome. Metformin has also been shown to alleviate weight gain associated with antipsychotic medication. Metformin has recently been extensively studied and emerging evidence suggests metformin decreases hepatocyte triglyceride accumulation in NAFLD and prevents liver tumorigenesis. Interestingly, studies have also shown metformin reduces visceral fat, suppresses white-adipose-tissue (WAT) extracellular matrix remodeling, and inhibits obesity-induced inflammation. However, clinical evidence for using metformin to treat NAFLD, cancer, metabolic syndrome, or to prevent hepatocellular carcinoma in NAFLD patients is lacking. This review therefore addresses the potential beneficial effects of metformin on NAFLD, its role in protecting against cardiac ischemia–reperfusion (I/R) injury, atherosclerosis, glucotoxicity, and lipotoxicity induced oxidative and ER stress in pancreatic β-cell dysfunction, as well as its underlying molecular mechanisms of action.

## 1. Introduction

Metformin, a guanidine derivative that was initially extracted from the plant *Galega officinalis* (French lilac) has been used as a glucose-lowering medication in humans for more than 60 years [1]. Metformin has proven to be safe and is highly cost-effective. Unlike other antidiabetic drugs, when used as a monotherapy, metformin does not have hypoglycemic side effects and has a favorable effect on body weight [2]. According to the American Diabetes Association (ADA) guidelines, of all the antidiabetic medications, metformin is now the recommended first-line treatment for type 2 diabetes (T2D) [3]. A reduction in hepatic glucose production has been established as the primary contribution to the glucose-reducing effect of metformin; however, the mechanism of action of this old drug is still not fully understood [1,4]. The United Kingdom Prospective Diabetic Study (UKPDS) observed reduced mortality rates in patients using metformin [5]. Furthermore, metformin is one of the few drugs that significantly reduce macrovascular events in diabetes patients when compared to other antidiabetic medications [2,5]. In addition, it may also be effective in a number of other applications, such as treating cancer, obesity, nonalcoholic fatty liver disease (NAFLD), polycystic ovary syndrome (PCOS), and metabolic syndrome [4]. Furthermore, antiaging and bone-protective effects, especially in the first stages of rheumatic arthritis (RA), have also been shown very recently [6,7]. Emerging evidence suggests metformin may have many other benefits, including reducing endoplasmic reticulum (ER) and oxidative stress, as well as anti-inflammatory properties [8,9]. The diverse effects of metformin could be the result of the interaction of metformin with numerous enzymes, including mitochondrial electron transport chain complex I and AMP-activated protein kinase (AMPK) [10]. This review will address the currently available evidence on the effects of metformin on NAFLD, inflammation, and cardiovascular and pancreatic β-cell dysfunction.

## 2. Metformin Improves Nonalcoholic Fatty Liver Disease

NAFLD includes a wide range of liver pathologies, ranging from steatosis, nonalcoholic steatohepatitis (NASH), cirrhosis, and may progress to hepatocellular carcinoma (HCC) [11,12]. Obesity, insulin resistance, and T2D in particular are major contributors to the development of NAFLD and NASH. NASH can therefore be called “diabetic hepatopathy” [13]. Unfortunately, the prevalence of NAFLD and NASH are increasing due to the epidemics of obesity and diabetes. There is currently no established therapy for NAFLD/NASH. The only interventions that have proven to be effective are weight loss and physical activity [14]. The benefits of metformin in inhibiting hepatic gluconeogenesis, modifying hepatic fatty acid metabolism (including inhibition of adipose-tissue lipolysis), elevating of fatty acid oxidation, as well as inhibiting lipogenesis and enhancing insulin sensitivity are well-established [4,15]. These favorable effects of metformin on liver histology in patients with NAFLD/NASH have been reported in several recent studies [16,17,18]. In addition, improvement in serum Alanine transaminase (ALT) has also been reported [19]. However, clinical evidence for using metformin to treat NAFLD is still lacking.

### 2.1 Metformin Decreases Hepatocyte Triglyceride Accumulation and Plasma Triglyceride

Hepatocyte triglyceride (TG) accumulation is a hallmark of NAFLD. Metformin suppresses hepatic accumulation of TG induced by a high-fat diet (HFD) in vivo and in vitro [20]. Furthermore, TG accumulation in HepG2 cells induced by hyperglycemia and hyperinsulinemia were attenuated by metformin (3 mM) treatment [20]. Finally, hepatic and plasma TG in obese mice were also reduced by metformin treatment in a dose-dependent manner [21,22].

**Figure 1:**
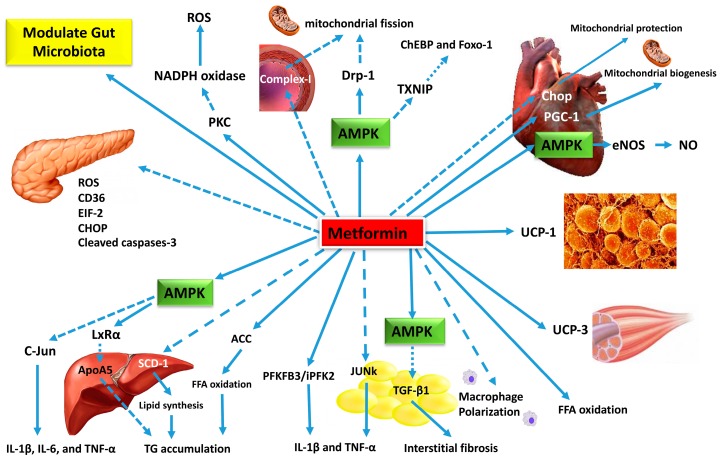
Potential underlying molecular mechanisms of action of metformin on NAFLD, atherosclerosis, oxidative stress, and pancreatic β-cell dysfunction (dotted arrows: inhibition, solid arrow: activation).

The underlying mechanism of hepatic and plasma TG reduction following metformin treatment has been reported by several studies. Apolipoprotein A5 (ApoA5) plays a key role in regulating TG metabolism, particularly in the formation of lipid droplets in hepatocytes [22]. Significant elevation of plasma ApoA5 levels has been positively correlated with hepatocyte TG in obese mice [22]. Four weeks of treatment with metformin (50 or 100 mg/kg) also reduces hepatocyte ApoA5 expression in obese mice [21,22]. Interestingly, hepatic TG reduction induced by ApoA5 knockdown is further reduced by metformin treatment [22], suggesting the involvement of ApoA5 in metformin’s effect. Liver X receptor α (LXRα) is a transcription factor that plays an important role in the regulation of ApoA5 mRNA [22]. Metformin-induced downregulation of ApoA5 is associated with increased phosphorylation of cellular AMPK, a metabolite-sensing protein kinase, and LXRα in both Hep-G2 cells and obese-mice hepatocytes [20,22]. However, inhibiting AMPK using inhibitors or knocking down of LXRα through siRNA significantly diminished the downregulation of cellular ApoA5 expression induced by metformin treatment [22]. Taken together, metformin reduces hepatic TG accumulation through the inhibition of hepatic ApoA5 synthesis, which is partially mediated through the AMPK/LXRα signaling pathway (Table 1 and Figure 1).

Inhibition of hepatocyte lipid synthesis by metformin may contribute to the reduction of TG accumulation [20,23]. Stearyl-coenzyme A desaturase 1 (SCD1) is an enzyme that participates in lipid de novo synthesis and catalyzes saturated fatty acids to form monounsaturated fatty acids [20]. Knockdown of SCD1 decreases TG levels in AML12 cells [20]. Metformin has been observed to decrease the expression of SCD1; furthermore, the effect of metformin on decreasing TG accumulation is reduced when SCD1 is overexpressed in HepG2 cells [20]. Thus, inhibition of SCD1 partially contributes to the effect of metformin in reducing hepatic lipid synthesis.

The underlying mechanisms of metformin in lowering plasma triglycerides were studied in APOE*3-Leiden CETP mice, which offer a well-established mouse model of humanlike lipoprotein metabolism [24]. Metformin (200 mg/kg) significantly reduced plasma TG levels in this mouse model, reduced production of hepatic very-low-density lipoprotein (VLDL)-TG, and lowered hepatic lipid composition [24]. Interestingly, metformin selectively elevated glycerol tri[(3)H]oleate-labeled VLDL-like emulsion-particle clearance by brown adipose tissue (BAT), suggesting elevation of BAT VLDL-TG clearance induced by metformin significantly contributed to the reduced plasma TG levels in mice [24] (Table 1).

### 2.2. Combination Therapy Increases the Therapeutic Efficacy in Treating NAFLD

It has been established that Sirtuin-1 (Sirt1), AMPK, and endothelial nitric oxide synthase (eNOS) regulate energy metabolism in the liver [26,27,28]. Metformin has been known to promote the activity of eNOS by activating the AMPK signal pathway [29]. L-leucine, an allosteric Sirt1 activator, combined with low doses of metformin or sildenafil were able to reverse mild NAFLD in mouse models via the AMPK-eNOS-Sirt1 pathway [26]. A combination of sildenafil–metformin–leucine has been shown to reduce inflammatory markers in vitro, increase hepatic fatty acid oxidation, and reduce lipogenic-gene expression [26]. High-fat-diet-induced increases of alanine aminotransferase (ALT), transforming growth factor-β (TGF-β), interleukin 1 β (IL1β), tumor necrosis factor α (TNFα), and hepatic collagen expression were significantly reduced by a combination of these three medications. Importantly, hepatocyte ballooning and triglyceride accumulation were completely reversed after the combination of sildenafil–metformin–leucin therapy [26].

The combination of L-cysteine and metformin have also been shown to suppress HFD-induced insulin resistance in streptozotocin-induced T2D rats [30]. Significant reductions in serum free fatty acids (FFAs), oxidative stress and inflammatory markers, caspase-3, and cytochrome c levels were detected with metformin monotherapy in T2D rats compared to controls, and a combination of L-cysteine and metformin therapy led to even greater improvements in these domains [30].

Greater improvements of liver histology, as well as reductions of oxidative stress and plasma levels of inflammation markers, including C-reactive protein (CRP), TNF-α, and interleukine-6 (IL-6) were also seen in T2D rats treated for two weeks with a combination of metformin and atorvastatin when compared to metformin monotherapy [10]. Taken together, these combination therapies were able to increase the therapeutic efficacy of metformin in the treatment of NAFLD and NASH better than monotherapy in animal models. However, clinical studies examining the therapeutic efficacy of combination therapies are still lacking.

### 2.3. Metformin Prevents High-Fat-Diet-Induced Liver Tumorigenesis

Hepatic steatosis is a key risk factor in the development of HCC. Metformin treatment has been found to prevent HFD-induced liver tumorigenesis, and the underlying mechanism is likely associated with the suppression of adipose-tissue inflammation [12]. However, metformin (250 mg/kg/d) failed to protect against HFD-induced liver tumorigenesis in mice following the development of NAFLD [12]. This suggests early intervention with metformin to suppress liver-fat accumulation prior to the onset of NAFLD, thus delaying adipose inflammation, may help to prevent HFD-induced liver tumorigenesis [12]. However, clinical evidence supporting metformin’s ability to modify the risk of HCC in NAFLD/NASH patients has not been reported. The effects of metformin on NAFLD and potential mechanisms involved are summarized in Table 1.

## 3. Metformin Has a Beneficial Effect on Energy Metabolism and WAT Remodeling

Metformin is known to have a beneficial impact on weight loss and energy metabolism [31]. Visceral fat mass is an important factor that contributes to the development of metabolic syndrome, and metformin has been shown to significantly reduce visceral fat mass in mice [31]. Furthermore, a clinical study conducted on the Chinese population demonstrated that metformin monotherapy for 24 weeks resulted in significant weight loss, reductions in body fat mass, and better blood-glucose control among T2D patients with NAFLD [32]. Additionally, body composition, measured using dual-energy X-ray absorptiometry, indicated that trunk, limb, android, and gynoid fat mass were reduced, and this was associated with reduced serum alanine-aminotransferase concentrations and serum aspartate-aminotransferase concentrations [32]. It is recognized that metformin-induced intracellular TG lipolysis and fatty acid oxidation contribute to its beneficial effect on energy metabolism [24]. However, the precise underlying mechanisms of these beneficial effects on energy metabolism and body weight have not been fully elucidated and require further exploration.

### 3.1. Metformin Reduces Visceral Fat Mass Through Adaptive Thermogenesis

Metformin treatment for two weeks significantly decreased baseline respiratory quotient (RQ) and increased postprandial RQ in healthy subjects, as well as patients with T2D [31,33]. BAT is an important regulator of energy metabolism and one study has shown that the expression of uncoupling protein-1 (UCP-1) in BAT and uncoupling protein-3 (UCP-3) in skeletal muscle were upregulated after metformin treatment (1500 mg/day) [31]. Furthermore, uptake of the murine interscapular BAT depot was detected following injection of [11C]-metformin, confirming metformin’s ability to target BAT in vivo [24,34].

Metformin has also been shown to attenuate weight gain induced by antipsychotic medication. Significant weight gain is a common side effect of olanzapine, a first-line treatment for schizophrenia. One study has shown that, after two weeks of metformin treatment, WAT accumulation and weight gain induced by olanzapine were significantly attenuated in patients [35]. Additionally, metformin salvaged BAT that would have been lost due to olanzapine treatment [35]. This evidence further indicates that upregulation of adaptive thermogenesis is a mechanism by which metformin reduces visceral fat. Additionally, gene-expression analysis showed metformin was able to modify the expression of multiple key energy expenditures [35]. Hence, metformin may reduce visceral fat and improve energy metabolism by upregulating adaptive thermogenesis [31] (Table 2 and Figure 1).

### 3.2. Metformin Suppresses Interstitial Fibrosis in Adipose Tissue

Interstitial fibrosis in WAT impairs adipocyte plasticity and exacerbates abnormal extracellular-matrix (ECM) remodeling, which has been recognized as an indicator of metabolic dysregulation in obesity [36]. In obese human subjects, WAT fibrosis is associated with AMPK inactivation, adipocyte apoptosis, and activation of TGF-β1 signaling, which is also known to play a central role in the pathogenesis of liver inflammation and fibrosis [36]. Metformin (250 mg/kg/d) significantly suppressed the expression of the fibrotic gene collagen cross-linking regulator lysyl oxidase (LOX), and reduced collagen deposition in adipocytes in both ob/ob mice and HFD-induced obese mice [36]. Metformin was unable to inhibit TGF-β1 and fibrogenesis when the dominant negative AMPK was expressed in primary cells of the stromal vascular fraction [36]. This evidence suggests that metformin reduces ECM remodeling in WAT through the inhibition of TGF-β1 signaling, which is AMPK-dependent [36]. The beneficial effects of metformin on energy metabolism and WAT remodeling are summarized in Table 2.

## 4. Metformin Suppresses Inflammation

### 4.1. Metformin Suppresses Adipocyte Inflammation

Reduction of adipose inflammation has been shown to contribute to the ability of metformin to improve obesity-associated metabolic dysregulation [37]. However, the underlying mechanism is still not well-understood. Adipose-tissue macrophages (ATMs), which constitute 5% of adipose tissue in a lean state, play an important role in removing dead adipocytes [38]. ATMs are significantly increased in both humans and mice during obesity [39] with a concomitant polarization toward the M1 phenotype (proinflammatory), which is associated with the development of metabolic syndrome [40]. One recent study indicates metformin reduces proinflammatory-cytokine production through inhibition of M1 macrophages and an elevation of the M2 (anti-inflammatory) macrophages [41]. Hence, the anti-inflammatory effect of metformin may be related to activation of anti-inflammatory macrophage polarization.

Suppression of lipopolysaccharide (LPS)-induced phosphorylation of Jun N-terminal Kinase (JNK) p46 and reduction of cytokine release, including interleukin-1β (IL-1β) and TNFα, after metformin (50 µM) treatment has also been reported in differentiated 3T3-L1 in vitro [37]. Metformin also increased expression of 6-phosphofructo-2-kinase/fructose-2,6-bisphosphatase (PFKFB3/iPFK2), but failed to inhibit LPS-induced inflammatory response in adipocytes when PFKFB3/iPFK2 was knocked down [37], thus suggesting LPS-induced inflammation is inhibited by metformin through PFKFB3/iPFK2 signaling.

### 4.2. Metformin Suppresses Obesity Induced Inflammation in Liver and Macrophages

Findings have been inconsistent regarding the anti-inflammatory effect of metformin on the liver during obesity. Short-term treatment (10 days) with metformin suppressed accumulation of lipids in the livers of obese mice, but induced inflammatory markers. Elevation of cytokine content in hepatocytes, including IL-1β, TNF-α, IL-6, MCP-1, and IFN-γ, as well as the concentration of IL-1β and IL-6 in a hepatocyte culture medium, was detected after short-term metformin treatment [31]. In contrast, long-term (four-week) treatment with metformin (150 mg/kg/d) was found to reduce inflammation in the liver of HFD-fed obese mice [15,32]. Similar to the findings in adipocyte tissue, metformin decreased the phosphorylation of c-JNK-1, reduced fat deposition as well as hepatocyte-proinflammatory cytokines in association with enhanced AMPK phosphorylation, and decreased fat deposition after long-term treatment with metformin [15,32]. Hence, the anti-inflammatory effect of metformin in the liver may require longer duration of treatment to achieve positive results.

Bone marrow-derived macrophages have also been assessed, and metformin partially suppressed LPS-induced phosphorylation of JNK1 and nuclear factor kappa B (NF-κB) p65, along with the reduction of proinflammatory cytokines in these cells [15,32]. Metformin has also been shown to lower the levels of IL-1β, IL-6, and TNF-α stimulated by LPS in a macrophage-culture medium [31].

In summary, reduction of the obesity-induced inflammatory response following metformin treatment may act through different mechanisms in different tissues. Multiple pathways are involved in the anti-inflammatory effect of metformin in adipose tissue, including modification of macrophage polarization towards an M2 phenotype, inhibition of the c-Junk pathway, and upregulation of PFKFB3/iPFK2 (Table 3). The anti-inflammatory effects of metformin in hepatocytes and macrophages may act through inhibition of the JNK pathway, but longer duration of treatment may be required to achieve therapeutic effects in the liver.

## 5. Cardiovascular Protective Effects of Metformin

It is well established that T2D patients have a remarkably higher risk of developing myocardial infarction (MI) and stroke than subjects without diabetes [33]. Although current antidiabetic medication is highly effective in treating hyperglycemia, T2D remains a high risk factor for cardiovascular disease (CVD). Furthermore, CVD-related morbidity and mortality do not benefit from intensive glycemic control [43,44].

### 5.1. Cardiovasclular Protective Effects of Metformin Have Been Shown in Clinical Trials

Results of several clinical trials suggest a cardiovascular protective effect of metformin in individuals with CVD [45,46,47]. UKPDS revealed reduced macrovascular complications that were independent from the glucose-lowering effect of metformin [5]. The risk of developing nonfatal MI in diabetes patients treated with metformin was reduced by 39% [5]. Importantly, the protective effects of metformin were observed even in the 10-year post-trial monitoring in patients who survived to the end of the UKPDS [48]. Another recent large double-blind randomized trial evaluated the cardiometabolic effects of metformin in overweight or obese type 1 diabetes adult patients who had high CVD risk. Reductions in body weight, LDL-cholesterol, and atherosclerosis progression, based on carotid artery intima-media thickness (a marker of CVD) were observed in that population [49]. A recent cohort study of older U.S. veterans with T2D also showed that metformin reduced CVD events among individuals with T2D [50]. These findings indicate the potential role of metformin for decreasing CVD risk, and evidence suggests that a combination therapy of metformin with statins has an even more favorable effect on CVD comorbidity in T2D patients [10].

### 5.2. Metformin Protects against Cardiac Ischemia-Reperfusion Injury and Development of Heart Failure

Acute MI is a major cause of debilitation and death worldwide. The reperfusion process, which typically occurs in patients presenting with an acute ST-segment elevation MI (STEMI), leads to further cardiomyocyte injury (myocardial ischemia reperfusion injury (IRI)) [51]. Unfortunately, there is still no effective therapy for IRI [51]. A reduction of infarct size and improved survival rates following MI in human subjects and animal models were observed after metformin treatment [52]. Even a single low-dose (125 µg/kg) therapy in nondiabetic and diabetic mice has demonstrated a protective effect after MI [52]. Several studies have suggested that metformin protects against cardiac IRI through the activation of AMPK [52,53,54]. Activation of AMPK promotes glycolysis and protects myocyte viability through closure of the mitochondrial permeability transition pore (mPTP), preventing the mPTP from opening and rupturing [52]. This effect is mediated by increased phosphorylation of eNOS, resulting in nitric oxide (NO) production. This evidence suggests metformin protects the heart against IRI through AMPK–eNOS signaling [52,53]. Finally, metformin has been observed to reduce myocardial injury after ischemia through restoration of depleted PGC-1α levels and increased mitochondrial biogenesis [53].

The beneficial effects of metformin on the heart have been observed in patients with and without heart failure [54]. Decreased left-ventricular dilatation and improvement of left-ventricular ejection fraction were detected in MI patients after twelve weeks of metformin treatment in subjects without diabetes, and is associated with decreased atrial natriuretic peptide [54]. Thus, metformin may attenuate cardiac remodeling and slow heart-failure development post-MI [54].

### 5.3. Metformin Attenuates ER Stress-Induced Mitochondrial Dysfunction in Myocardial Cells

ER stress is an important factor in mitochondrial dysfunction, which increases mitochondrial permeability via the opening of transition pores, and is known to contribute to cardiac injury during ischemia–reperfusion [55]. Metformin (300 mg/kg body weight) prevented ER stress-induced mitochondrial dysfunction in myocardial cells treated with thapsigargin, and reduced C/EBP homologous protein (CHOP) content in the cytosol and nucleus of myocardial cells [55]. The concentration of metformin in the mitochondria and the ER was found to be dependent on membrane potential [56]. In summary, metformin reduced cardiac injury during ER stress through the protection of cardiac mitochondria and attenuation of CHOP expression (Table 4).

### 5.4. Metformin Exhibits Vascular Protective Effects

Endothelial dysfunction is an important factor in the development of atherosclerosis in T2D patients [57], and may occur even at very early stages of the disease [58]. Severity of vascular endothelial dysfunction has been correlated even with recently and newly diagnosed T2D patients [57]. An antiatherogenic effect of metformin has been shown in previous studies [59,60], and, importantly, improvement of endothelial dysfunction with metformin treatment in newly diagnosed T2D patients has been detected [57]. However, the underlying mechanisms are still unknown.

Hyperglycemia induces mitochondrial superoxide production, and inhibits the expression of dynamin-related protein (Drp1) and its translocation into mitochondria in endothelial cells [61]. Superoxide production and fragmentation of mitochondria were markedly suppressed after metformin therapy. Furthermore, suppression of atherosclerotic lesions was detected in streptozotocin (STZ)-induced diabetic ApoE^−/−^ mice following metformin treatment [61]. In contrast, the protective effects of metformin on Drp1 expression, oxidative stress, and atherosclerosis were ablated when AMPK-α2 was knocked out in diabetic ApoE^−/−^/AMPK-α2^−/−^ mice, suggesting metformin exerts antiatherosclerotic action in vivo via the AMPK-mediated blockage of Drp1-mediated mitochondrial fission [61].

Several biochemical pathways have been found to be involved in hyperglycemia-induced reactive oxygen species (ROS) production in endothelial cells [62]. Among these, PKC-dependent activation of NAD (P) H oxidase is one of the major sources [63]. Metformin (10 µM) has been observed to prevent hyperglycemia-induced oxidative stress through inhibition of the PKC-NAD(P)H oxidase pathway in cultured human endothelial cells [62]. Another important source of ROS production is mitochondrial complex I (NADH: ubiquinone oxidoreductase), which plays an essential role in mitochondrial respiration and oxidative phosphorylation [64]. One study has shown that metformin directly inhibits both isolated complex I in intact cells and purifiedcomplex I [64]. Inhibition of mitochondrial respiratory complex I by metformin is therefore suggested as one of the therapeutic targets as well as inhibition of mitochondrial ATP synthase [64].

Inhibition of atherosclerosis progression by metformin has also been demonstrated recently in an atherogenic diet-induced rabbit atherosclerotic model [65]. Reduction of macrophage infiltration, inhibition of TNF-*α*-induced monocyte adhesion, and reduced inflammatory cytokine release from macrophages in endothelial cells were observed in the study [65].

Thioredoxin-interacting protein (TXNIP) regulates the cellular redox state and impairs endothelial function [66]. Two transcription factors, carbohydrate response element-binding protein (ChREBP) and forkhead box O1 (FOXO1), recruit the TXNIP promoter. Induction of TXNIP expression is detected after high glucose exposure in primary human aortic endothelial cells [66]. Metformin (final dose of 150 mg/kg/day) was able to attenuate hyperglycemia-induced TXNIP expression and reduce the nuclear entry rate of ChREBP and FOXO1 [66]. However, inhibition of AMPK partially diminished these protective effects. Hence, metformin protects against high glucose-induced endothelial cell dysfunction and exerts vascular protective effects by inactivating both ChREBP and FOXO1, a mechanism that is partially dependent on AMPK activation [66].

In conclusion, the role of metformin in treating macrovascular complications in patients with T2D is well-established. Emerging evidence indicates metformin inhibits atherosclerosis progression and improves endothelial dysfunction through multiple pathways, which includes reducing Drp1-mediated mitochondrial fission and inactivating both ChREBP and FOXO1 in an AMPK-dependent manner. Endothelial mitochondria are likely a major target of metformin, and through its inhibition of respiratory complex I, PKC-NAD (P) H oxidase, and ATP synthase, metformin is able to exert vascular protective effects. These cardiovascular protective effects of metformin, and the potential mechanisms involved, are summarized in Table 4.

### 5.5. Metformin Suppresses Angiotensin II-Induced ER Stress and Hypertension

Antihypertensive effects have been observed in diabetic patients taking metformin [67]; however, the underlying mechanism is unclear. One possible mechanism is the inhibition of Angiotensin II-induced ER stress through AMPK activation [67]. Expression of ER stress markers in Angiotensin II-infused wild-type (WT) mice were significantly suppressed by metformin (300 mg/kg) treatment; however, metformin lost its protective effect in reducing ER stress makers in AMPKα2-deficient mice [67]. It is therefore likely that metformin inhibits angiotensin II-induced ER stress in vascular smooth muscle cells and suppresses angiotensin II-induced hypertension by activating AMPKα2.

In summary, metformin has demonstrated cardiovascular protective effects via reduction of cardiac IRI, attenuation of cardiac remodeling, and inhibition of angiotensin II-induced ER stress in vascular smooth muscle cells.

## 6. Metformin Improves Dyslipidemia

Dyslipidemia, or diabetic dyslipidemia (dyslipidemia in T2DM patients), is an abnormal lipid metabolism that is characterized by elevation of plasma TG, low-density lipoprotein (LDL-C), and reduced plasma levels of high-density lipoprotein cholesterol (HDL-C) [68]. Due to their effective LDL-C-lowering effects, statins are recommend as the first-choice treatment for diabetic dyslipidemia [10]. High concentrations of insulin are attributable to the dysregulation of intestinal lipoprotein metabolism commonly seen in T2D patients [69]. Metformin treatment (2300 mg/day) was found to decrease intestine-derived TG-rich lipoproteins in T2D patients, reducing plasma chylomicrons by 50%, and chylomicron-remnant lipoprotein fractions  by 20% [70], suggesting metformin is able to improve intestinal lipoprotein metabolism. Metformin has been found to affect both intestinal and liver tissues resulting in decreased plasma triglycerides, LDL-C, and total cholesterol. However, metformin’s effects on lipid metabolism seems to be localized to the intestine [10].

A slight improvement of intestinal lipid homeostasis was observed in obese T2D patients treated with metformin in association with a decrease in mRNA expression of sterol regulatory element-binding protein 1 (SREBP-1c), ACC1, and Apo A-IV (involved in the secretion of chylomicrons) [69]. SREBP-1c is able to upregulate enzymes involved in de novo fatty acid synthesis, such as acetyl-CoA carboxylase (ACC1) and fatty acid synthase (FAS). However, insulin upregulates the expression of SREBP-1c, though it can be inhibited by AMPK. In conclusion, metformin, which improves intestinal lipoprotein metabolism and inhibition of SREBP-1C, which reduces fatty acid synthesis, may contribute to this beneficial effect in diabetic dyslipidemia.

## 7. Metformin Improves Pancreatic β-Cell Function

Both β-cell dysfunction and insulin resistance are characteristics of T2D. It is known that oxidative stress and inflammation result from hyperglycemia and eventually lead to impaired insulin secretion and increased apoptosis in β-cells [71]. In addition, long-term exposure to FFA results in suppressed glucose-stimulated insulin secretion (GSIS) and reduced insulin biosynthesis [72], eventually leading to adaptive decline of β-cell mass and/or function as a compensatory response to insulin resistance. Chronic glucose and fatty acid exposure eventually result in β-cell failure and the development of diabetes [72,73].

### 7.1. Metformin Reduces Compensatory Pancreatic β-Cell Hyperplasia

The effect of metformin on high-glucose-induced pancreatic β-cell hyperplasia remains controversial. HFD-triggered adaptive pancreatic β-cell replication was suppressed by eight weeks of metformin treatment [73]. However, after sixty weeks of HFD feeding, increased β-cell mass was not suppressed [73]. High-glucose-induced β-cell proliferation was also inhibited by metformin in both islets and INS-1 cells [73]. However, the underlying mechanism of metformin on reducing pancreatic β-cell hyperplasia is still unknown. In summary, metformin can directly suppress β-cell proliferation induced by HFD and high glucose.

### 7.2. Metformin Protects Pancreatic β–Cells against Glucotoxicity-Induced Oxidative and ER Stress

It is well established that glucotoxicity-induced oxidative and ER stress are pivotal in the development of β-cell dysfunction. FFA uptake is increased via induction of cluster determinant 36 (CD36), a fatty acid transporter, while insulin and pancreatic duodenal homeobox1 (Pdx1) mRNA expression are suppressed after high glucose exposure in INS-1 cells and isolated rat islets [74]. Intracellular ROS production and CD36 expression, induced by high glucose, were significantly inhibited after metformin (0.5 mM) treatment. In addition, CD36 activation by sulfa-*N*-succinimidyl oleate (SSO) significantly decreased the apoptotic response in high glucose-treated INS-1 cells [74]. In conclusion, metformin is able to protect against glucotoxicity-induced ROS production and inhibits the CD36-mediated free fatty acid influx in pancreatic β-cells.

### 7.3. Metformin Improves Chronic Exposure of Fatty Acid-Induced Pancreatic β-Cells Dysfunction

It has been established that prolonged exposure to palmitate impairs GSIS and contributes to β-cell dysfunction. Human islet cells in culture for seven days with palmitate showed a reduction of p-AMPK and a significant elevation of phosphorylated eukaryotic initiation factor-2 (p-EIF2α), CHOP, and cleaved caspase 3, but their levels were normalized in the presence of 25 µM metformin [75]. This suggests metformin is able to improve chronic fatty acid exposure-induced pancreatic β-cell dysfunction. This suggests metformin is able to improve pancreatic β-cell function following chronic fatty acid exposure, as well as protect pancreatic β-cells against glucotoxicity and lipotoxicity-induced oxidative and ER stress.

## 8. Metformin Modulates Gut Microbiota

Gut microbiota structure is altered with HFD, and emerging evidence suggests that gut microbiota are an important factor in mediating the development of metabolic syndrome and T2D [76,77]. Forty-six gut microbes have been found to be significantly changed after 30 days of treatment with metformin (200 mg/kg body weight) in healthy mice [77]. Particularly, the diversity of gut microbiota was significantly reduced [76,77] and the introduction of *Akkermansia* spp. into the gut of these diet-induced obese mice improved glucose homeostasis. The modulation of the gut microbiota may be one of the mechanisms contributing to the antidiabetic effects of metformin [76].

## 9. Conclusions

Metformin, an old drug with magical roles, has drawn much attention in recent years due to its newly recognized beneficial effects. Metformin has diverse effects through its action on different tissues including, but not limited to, liver, adipose, skeletal muscle, intestine, brown fat, heart, vascular, pancreas, and bones (as shown in Figure 1). These diverse effects may due to the action of metformin on several enzymes located in the mitochondria and ER, as AMPK is one of the primary therapeutic targets in liver, cardiovascular, and adipose tissue.

Metformin inhibits inflammation in liver tissue, macrophages, vasculature, and adipocytes. Metformin also increases energy metabolism by upregulating adaptive thermogenesis, inhibiting lipid synthesis, and promoting fatty acid oxidation. Furthermore, metformin prevents glucotoxicity-induced pancreatic β-cell dysfunction via reduction of ROS and CD36-mediated FFA influx. Metformin also significantly reduces macrovascular events and diabetes-related mortality, and has been shown to protect against cardiac IRI, and to delay the development of heart failure through mechanisms not completely understood. Finally, it reduces microbial diversity, which may also contribute to its beneficial effects.

Metformin has been suggested for a number of potentially new clinical applications. However, there is little clinical evidence that supports all these new applications. For example, its long-term clinical outcomes in patients with NASH, particularly in reducing the risk of HCC in patients with NAFLD/NASH, are unclear. Future large-scale clinical trials with longer durations to assess the effectiveness of metformin in reducing HCC risks are needed. Liver biopsy is still the current gold standard for diagnosis of NASH since reliable noninvasive tests of histological or biochemical markers are lacking. Although combination therapies have shown more favorable effects in treating NAFLD than metformin monotherapy in animal models, more clinical studies are needed to address therapeutic efficacy before its clinical application. In addition, whether combination therapy would reverse mild NAFLD is still unclear.

Hence, although there are many putative applications of metformin in an enormous spectrum of diseases, many mechanisms remain to be elucidated. More clinical evidence is needed before the therapeutic application of metformin can be extended to treat those diseases outside of diabetes.

## Figures and Tables

**Table 1:** Metformin improves nonalcoholic fatty liver disease (NAFLD).

Tissue	Metformin Effects and Mechanisms	Subjects	Reference
Hepatocyte	Improvement in hepatocellular ballooning	Human and animal	[16,25]
	Reduced TG accumulation	Ob/ob mice, HepG2 cell	[20,21,22,23]
	ApoA5 decreased	Ob/ob mice, HepG2 cell	[21,22]
	Phosphorylation of AMPK increased	Ob/ob mice	[20,21,22]
	LXRα increased	Mice, AML 12 cells	[22]
	SCD1 decreased	AML 12 cell, HepG2 cell	[20]
Plasma	Total cholesterol and TG reduced	APOE*3-Leiden CETP mice	[24]
	Hepatic VLDL-TG production reduced		
	BAT VLDL-TG clearance increased		

**Table 2:** Beneficial effects of metformin on energy metabolism and white-adipose-tissue (WAT) remodeling.

Tissues	Metformin Effects and Mechanisms	Subjects	Reference
BAT	UCP-1 increased	Mice	[24,31,34]
Skeletal muscle	UCP-3 increased	Mice	[31]
Adipocyte	Lipogenic markers reduced	Humans and mice	[31]
	Activation of AMPK increased		[31]
Stromal vascular fraction	TGF-β1 reduced	Humans and mice	[36]

**Table 3:** Metformin reduces inflammation.

Tissues	Metformin Effects and Mechanism	Subjects	References
Hepatocyte	Phosphorylation of C-JUNK-1 decreased	Obese mice	[23]
	AMPK activation increased	Obese mice	[23]
Adipocyte	PFKFB3/iPFK2 increased	3T3L-1 cell	[37]
Macrophages	IL-1β, IL-6 and TNF-α decreased	Obese mice	[42]
	Alteration of macrophage polarization	Mice	[41]

**Table 4:** Cardiovascular protective effect of metformin.

Tissue	Metformin Effects and Mechanism	Subjects	References
Vascular smooth muscle cells	Infarct size smaller	Sprague–Dawley rats	[52,54]
	Left-ventricular dilatation reduced	Diabetic rat	
	Left-ventricular ejection fraction improved	Human, diabetic rat	
	AMPK activation increased	Diabetic rats	[52,53,54]
	eNOS increased	Diabetic rats	[52,53]
	THAP-induced CHOP reduced	Mice	[55]
Aortic endothelial cell	TXNIP reduced	Human	[66]
	ChREBP decreased	Human	[66]
	FOXO-1 decreased	Human	[66]
	TXNIP decreased	Human	[66]
	AMPK increased	Human	[66]
	ER stress markers reduced	Human	
	Adhesion molecules reduced	Rabbit	[65]
	Inflammatory cytokines reduced	Rabbit	[65]
	Atherosclerotic plaques decreased	Rabbit	[65]
	Macrophage content in lesions reduced	Rabbit	[64]
	Mitochondrial Complex I suppression Inhibition of mitochondrial fission Inhibition of PKC-NAD(P)H oxidase	MiceHuman	[62]

## Abbreviations

ApoA5	Apolipoprotein A5
ACC	Acetyl-CoA carboxylase
AMPK	AMP-activated protein kinase
CD36	Cluster determinant 36
CHOP	C/EBP homologous protein
ChREBP	Carbohydrate response element-binding protein
FOXO1	Forkhead box O1
HCC	Hepatocellular carcinoma
HFD	High-fat diets
IL-1β	Interleukine-1 β
JNK	Jun N-terminal Kinase
LXRα	Liver X receptor α
NASH	Nonalcoholic steatohepatitis
NAFLD	Nonalcoholic fatty liver disease
PFKFB3/iPFK2	6-Phosphofructo-2-Kinase/Fructose-2,6-Bisphosphatase
SREBP-1	Sterol regulatory element-binding protein 1
SCD1	Stearyl-coenzyme A desaturase 1
TXNIP	Thioredoxin-interacting protein
TNFα	Tumor Necrosis Factor-α
TGF-β1	Transforming growth factor-beta1
UKPDS	United Kingdom Prospective Diabetic Study
